# The effect of an attachment‐oriented couple intervention for breast cancer patients and partners in the early treatment phase: A randomised controlled trial

**DOI:** 10.1002/pon.4613

**Published:** 2018-01-26

**Authors:** A. Nicolaisen, M. Hagedoorn, D.G. Hansen, H.L. Flyger, R. Christensen, N. Rottmann, P.B. Lunn, H. Terp, K. Soee, C. Johansen

**Affiliations:** ^1^ National Research Centre for Cancer Rehabilitation, Research Unit of General Practice, Department of Public Health University of Southern Denmark Odense C Denmark; ^2^ Center for Quality Region of Southern Denmark Middelfart Denmark; ^3^ Department of Health Psychology University Medical Center Groningen, University of Groningen Groningen The Netherlands; ^4^ Department of Breast Surgery Herlev University Hospital Herlev Denmark; ^5^ Department of Psychology University of Southern Denmark Odense M Denmark; ^6^ Department of Plastic Surgery and Breast Surgery Ringsted Hospital Ringsted Denmark; ^7^ Centre for Breast Surgery, Department of Plastic Surgery Odense University Hospital Odense Denmark; ^8^ Danish Cancer Society Research Center, Survivorship Danish Cancer Society Copenhagen Denmark; ^9^ Oncology Clinic, Finsen Centre Rigshospitalet, University of Copenhagen Copenhagen Denmark

**Keywords:** attachment, breast cancer, couples, distress, dyadic coping

## Abstract

**Objective:**

Patients and partners both cope individually and as a dyad with challenges related to a breast cancer diagnosis. The objective of this study was to evaluate the effect of a psychological attachment‐oriented couple intervention for breast cancer patients and partners in the early treatment phase.

**Methods:**

A randomised controlled trial including 198 recently diagnosed breast cancer patients and their partners. Couples were randomised to the Hand in Hand (HiH) intervention in addition to usual care or to usual care only. Self‐report assessments were conducted for both patients and partners at baseline, postintervention (5 months), and follow‐up (10 months), assessing cancer‐related distress, symptoms of anxiety and depression, and dyadic adjustment. Patients' cancer‐related distress was the primary outcome.

**Results:**

Cancer‐related distress decreased over time in both patients and partners, but the intervention did not significantly affect this decrease at postintervention (P = .08) or follow‐up (P = .71). A significant positive effect was found on dyadic adjustment at follow‐up for both patients (P = .04) and partners (P = .02).

**Conclusions:**

There was no significant effect of the HiH intervention cancer‐related distress. The results suggest that most couples can cope with cancer‐related distress in the context of usual care. However, the positive effect on dyadic adjustment implies that the HiH intervention benefitted both patients and partners. Future studies should investigate how to integrate a couple focus in usual cancer care to improve dyadic coping in the early treatment phase.

## 1 INTRODUCTION

Breast cancer (BC) is a life‐threatening disease, and patients are at increased risk of experiencing individual distress (including symptoms of anxiety and depression) at some point or continually during time of diagnosis and active treatment.^1^, ^2^, ^3^, ^4^, ^5^ Patients in an intimate relationship usually regard their partner as the main source of support throughout the cancer trajectory.^6^, ^7^ However, partners themselves are affected emotionally and experience challenges in how to support the patient.^8^ Patients' and partners' levels of distress may be affected not only by challenges associated with cancer diagnosis and treatment but also by perceived spousal support or lack thereof. The communication within the couple influences the couple's functioning.^9^, ^10^ Challenges that are not adequately coped with within the couple may increase levels of dyadic distress.^11^, ^12^

Three systematic reviews with 37 couple interventions for cancer patients and partners found significant, small to moderate effect sizes regarding psychological, physical, and relationship outcomes for both patients and partners.^13^, ^14^, ^15^ However, all authors concluded that the results were influenced by conceptual and methodological limitations of the intervention studies, such as no specified theoretical framework, small sample sizes, high attrition rates, and limited use of intention‐to‐treat analysis.

The Hand in Hand (HiH) randomised controlled trial (RCT) for couples coping with BC evaluates the effects of a psychological couple intervention in the early treatment phase addressing some of the methodological limitations seen in previous couple intervention studies. The theoretical framework is attachment theory, providing an explanation of how attachment behaviour and attachment style may influence the exchange of support within couples and their adjustment to BC.^16^, ^17^, ^18^, ^19^

### 1.1 Aim

The aim of this adequately powered study was to evaluate the effect of the HiH intervention for BC patients and their partners in addition to usual care compared to usual care only. The primary outcome was cancer‐related distress for patients at postintervention (T2) being regarded as the primary burden for both patients and partners. Secondary outcomes were cancer‐related distress for partners, symptoms of anxiety and depression, and dyadic adjustment for both patients and partners.

## 2 MATERIAL AND METHODS

The HiH study is a multicentre RCT of 198 couples coping with newly diagnosed primary BC. Couples were randomised to usual care or the HiH intervention in addition to usual care. A more detailed description of the HiH study has been published.^20^ The study was approved by the Danish Data Protection Board (No: 2012‐41‐0392) and the Regional Scientific Ethics Committee for Southern Denmark (No: S‐20110100).

### 2.1 Participants

Eligible patients were women newly diagnosed with primary BC, who were ≥18 years, cohabited with a male partner, had no previous cancer diagnoses, had received no neoadjuvant treatment, had no history of hospitalisation due to psychosis, were able to read and speak Danish, and were not referred to or consulting any of the trial psychologists. Partners had to be ≥18 years and be able to read and speak Danish.

### 2.2 Enrolment

Eligible patients were identified and informed about the project during their hospital admission in relation to primary surgery. Enrolment was conducted at 3 Danish breast surgery departments from October 2011 to December 2012 for centres 1* and 2,† and April 2012 to January 2013 for centre 3.‡ Consenting patients received additional information about the project by phone. If they consented to participate in the study, their partners were asked for verbal consent. Couples were randomised if completed questionnaires and signed consent forms had been returned.

Randomisation was stratified on centres, and each centre was block randomised. All except the independent statistician were blinded to block sizes and allocation sequence. Participants were for obvious reasons not blinded. Due to geographical reasons, it was not possible to randomise the psychologists to centres.

### 2.3 Control condition: usual care

Usual care at all 3 centres consisted of verbal and written information on normal psychological reactions in relation to a cancer diagnosis. It was distributed by the local clinical staff.

### 2.4 Intervention: HiH in addition to usual care

The HiH intervention consisted of 4 to 8 couple sessions led by a clinical psychologist up to 5 months after primary surgery. Attendance of both the patient and partner was required. The HiH intervention aimed to enhance dyadic adjustment through dyadic coping within the couples (eg, mutual understanding of attachment behaviour, perceived proximity and security, and creating new emotional experiences). The following issues should be addressed during couple sessions: couples' sense of attachment‐related security, level of individual emotional distress and needs, knowledge of and experiences with cancer, psychological disorders, former stress‐full life events, intimacy and sexual function, and other stressors. Enrolment implied 4 to 8 couple sessions, but the total number of couple sessions was decided by the couple and their allocated psychologist. All trial psychologists were experienced in working with therapeutic counselling of cancer patients and couples. Further details on the HiH intervention can be found in the published protocol article^20^ and Appendix S1.

### 2.5 Measurements

Data were obtained from a national database (time of primary surgery, T0) and self‐assessment questionnaires at preintervention (T1), postintervention 5 months after surgery (T2), and follow‐up 10 months after surgery (T3).

#### 2.5.1 Cancer‐related distress

The Impact of Event Scale (IES)^21^ assessed current subjective distress related to BC. The IES is a 14‐item scale with sum scores ranging from 0 to 70. Scores of 0 to 8 indicate no meaningful impact, 9 to 25 some impact, 26 to 43 a powerful impact, and ≥44 a severe impact. Cronbach's alphas were 0.89 to 0.92 for patients and 0.83 to 0.89 for partners.

#### 2.5.2 Symptoms of anxiety and depression

The Hospital Anxiety and Depression Scale^22^ is a 14‐item scale assessing feelings of anxiety and depressive symptoms in the past 7 days. Total scores of the subscales anxiety and depression range from 0 to 21 with >10 indicating a probable diagnosis, 8 to 10 indicating a possible diagnosis, and <8 low occurrence of anxiety and depression. Cronbach's alphas ranged from 0.78 to 0.87 for patients and 0.79 to 0.84 for partners.

#### 2.5.3 Dyadic adjustment

The Revised Dyadic Adjustment Scale^23^ assessed dyadic adjustment. The scale consists of 14 items. Total scores range from 0 to 69. Higher scores indicate greater dyadic adjustment measured by the degree of consensus, satisfaction, and cohesion in the relationship. Cronbach's alphas ranged from 0.77 to 0.93 for patients and 0.83 to 0.94 for partners.

#### 2.5.4 Therapeutic alliance

The “Bond” subscale from the Working Alliance Inventory—Short Revised assessed patients' and partners' perceptions of an affective bond between the psychologist and themselves.^24^ These items were added to the questionnaire at T2 for participants in the intervention group. The scores range from 0 to 28.

### 2.6 Additional support

At T2, all participants were asked if they had received any professional support and counselling (other than the intervention) from a doctor, nurse, psychologist, priest, social worker, or support group.

### 2.7 Demographic and medical variables

Breast cancer characteristics were obtained from the Danish Breast Cancer Group—clinical database.^25^ Cohabitation status and age were obtained from the Civil Registration System.^26^

### 2.8 Sample size

The required sample size was calculated based on a 7‐point difference in the change from T1 to T2 between the randomised groups on the IES Total measure. On the basis of prior intervention studies of BC patients and their partners, we estimated a mean of 27 at baseline with a standard deviation of 16 for patients.^27^, ^28^ A sample of 166 couples is sufficient to detect a relevant effect, with a power of 0.80 and an alpha of 0.05. Considering attrition rates reported in other couple intervention studies,^13^ we included 199 couples.

### 2.9 Statistical methods

Descriptive statistics were used to present demographic and disease‐related variables at baseline. We used linear regression adjusted for baseline scores of the respective outcome and chemotherapy to test the effect of the HiH intervention on cancer‐related distress, symptoms of anxiety and depression, and dyadic adjustment at T2 and T3. Further, we used linear regression analysis on the primary outcome adjusted for tumour size, type of operation, biological treatment, radiation, and nodal status. All analyses were modified intention‐to‐treat analysis. Effect sizes of differences between intervention and control group were calculated using Cohen *d*. Exploratory analyses investigated the effect of number of sessions and therapeutic alliance on cancer‐related distress for complete cases at T2 and T3 in the intervention group, using linear models adjusted for baseline scores.

Additionally, linear models were used to investigate interactions that could elucidate our findings on IES Total regarding the effect over time for initially distressed patients and partners. Interactions between group and time and between group and baseline distress were analysed.

## 3 RESULTS

### 3.1 Study population

**Figure 1:**
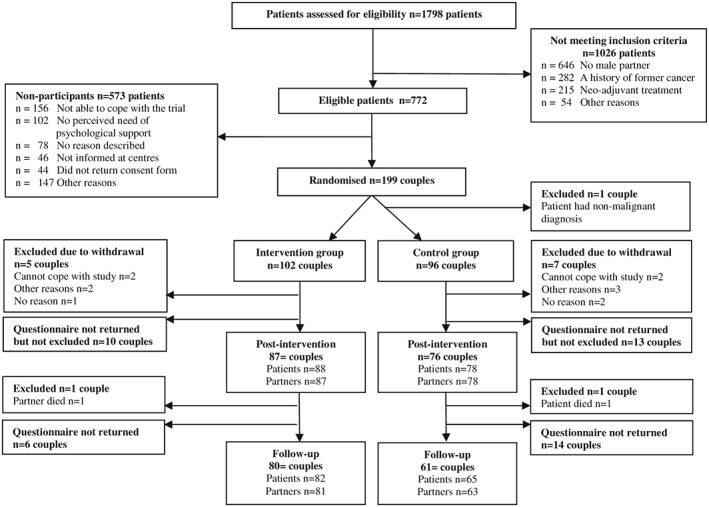
CONSORT flow diagram

Of 776 eligible couples, 198 (26%) were randomised into the intervention group (n = 102 couples) and the control group (n = 96 couples) (Figure 1). Baseline characteristics of enrolled patients and partners are shown in Table 1. A total of 166 patients at T2 and 147 patients at T3 completed the follow‐up questionnaires, resulting in a mean attrition rate from baseline to T3 of 26%. A total of 165 partners at T2 and 144 partners at T3 completed the follow‐up questionnaires, resulting in a mean attrition rate from baseline to T3 of 27%. Attrition was highest in the control group at T3 (37% compared to 20% in the intervention group). Dropouts and complete cases did not differ significantly in cancer‐related distress at baseline. Fifty‐three couples completed 4 to 8 sessions, 40 couples completed 1 to 3 sessions, and 9 couples did not complete any sessions.

**Table 1:** Sociodemographic, disease‐related, and treatment‐related characteristics of participants

		Intervention Group	Control Group
Sociodemographic data		N = 102 Couples	N = 96 Couples
		Mean (SD) range	Mean (SD) range
Age	F	54.2 (11) 27‐79	52.6 (10) 31‐75
	M	57.4 (12) 28‐92	56.4 (11) 35‐78
Relationship length in years		27.1 (15) 1‐60	25 (13) 2‐51
Education		N (%)	N (%)
Basic or high school	F	17 (17)	16 (17)
	M	15 (15)	16 (17)
Vocational education	F	35 (34)	39 (41)
	M	39 (38)	41 (43)
Higher education	F	50 (49)	41 (43)
	M	48 (47)	39 (41)
Disease‐related information		N (%)	N (%)
Tumour size			
Up to 20 mm		66 (65)	70 (73)
>20 mm		36 (35)	26 (27)
Lymph node involvement			
Yes		42 (41)	37 (39)
No		60 (59)	59 (62)
Type of surgery			
Mastectomy		30 (29)	20 (21)
Lumpectomy		72 (71)	76 (79)
Induced adjuvant therapy			
Chemotherapy		66 (65)	71 (74)
Radiation therapy		84 (82)	84 (88)
Hormone therapy		80 (78)	78 (81)
Trastuzumab		19 (19)	17 (18)

Abbreviations: F, females; M, males; SD, standard deviation.

On average, patients in the intervention group perceived a powerful impact of BC in the intervention group assessed by IES (mean, 26.28). Patients and partners in both groups scored relatively low on symptoms of anxiety and depression. Overall, dyadic adjustment increased in the intervention group, while it decreased in the control group.

### 3.2 Primary outcome

We found a significant positive effect of the intervention on patients' cancer‐related distress between the intervention and control group at T2 (*P* = .05), but after adjusting for baseline, the effect was nonsignificant (*P* = .08) with an effect size of −0.32 (Table 2). Adjusting for disease‐specific variables had no significant effect on the primary outcome.

**Table 2:** Study outcomes of patients and partners according to allocation status adjusted for baseline

		Baseline		Postintervention			Follow‐up		
		Mean (SD)		Mean (SD)			Mean (SD)		
		Intervention	Control	Intervention	Control	*P* value	Intervention	Control	*P* value
Cancer‐related distress									
IES Total^a^	F	26.3 (15.8) n = 101	24.5 (14.9) n = 94	20.0 (16.1) n = 88	21.6 (16.0) n = 77	.08	20.0 (15.4) n = 82	16.7 (13.7) n = 63	.71
	M	19.0 (11.7) n = 100	18.0 (10.9) n = 95	15.1 (11.6) n = 86	14.6 (10.6) n = 76	.99	15.0 (13.0) n = 81	12.8 (10.4) n = 63	.27
Symptoms of anxiety and depression									
HADS anxiety	F	5.9 (4.2) n = 101	6.3 (4.0) n = 94	5.6 (4.0) n = 88	5.4 (3.9) n = 77	.28	5.2 (4.1) n = 82	5.2 (3.3) n = 63	.75
	M	5.1 (3.6) n = 101	5.2 (3.3) n = 95	4.0 (3.3) n = 86	4.2 (3.4) n = 77	.77	4.1 (3.3) n = 81	3.8 (3.0) n = 63	.25
HADS depression	F	3.2 (3.6) n = 101 2.7 (2.9) n = 101	3.3 (2.9) n = 94 2.6 (2.5) n = 95	3.3 (3.7) n = 88	3.3 (3.2) n = 77	.92	2.6 (3.0) n = 82	2.6 (2.9) n = 63	.80
	M			2.7 (2.8) n = 86	2.3 (2.6) n = 77	.14	2.6 (3.0) n = 81	1.6 (2.0) n = 63	.01
Dyadic adjustment									
RDAS	F	49.8 (4.0) n = 102	49.7 (3.5) n = 94	50.4 (4.0) n = 88	49.5 (3.7) n = 76	.24	50.1 (4.0) n = 82	48.8 (3.4) n = 64	.04
	M	49.9 (3.7) n = 100	49.8 (3.5) n = 95	50.7 (3.9) n = 85	48.9 (8.4) n = 77	.10	50.2 (3.9) n = 76	48.8 (4.3) n = 62	.02

Abbreviations: F, female; M, male. HADS, Hospital Anxiety and Depression Scale; IES, Impact of Event Scale; RDAS, Revised Dyadic Adjustment Scale; SD = standard deviation.

a Primary outcome.

### 3.3 Secondary outcomes

There was no significant effect on cancer‐related distress for partners at T2 (*P* = .99) or for patients (*P* = .71) and partners (*P* = .27) at T3. Effect sizes varied from −0.06 to 0.14. There was no significant effect of the intervention on symptoms of anxiety and depression and dyadic adjustment at T2 for neither patients nor partners with effect sizes from 0.01 to 0.24. At T3, there was a negative effect for partners' symptoms of depression (*P* = .01) with an effect size of 0.36 and a significant effect on dyadic adjustment for both patients (*P* = .04) and partners (*P* = .02) with effect sizes of 0.28 and 0.37 compared to the control group.

### 3.4 Exploratory analyses

No interaction was found between level of cancer‐related distress and group that could indicate a larger effect for highly distressed patients at T2 (*P* = .93) or T3 (*P* = .38). In addition, we found no interaction between time and group that could indicate that the intervention had a differential effect on cancer‐related distress over time. Patients receiving 5 to 8 couple sessions had the largest decrease in total cancer‐related distress with a mean reduction of −16.5 at T2 and −10.3 at T3, compared to −5.9 at T2 and −5.2 at T3 when receiving 0 sessions, −4.0 at T2 and −4.0 at T3 when receiving 1 to 3 sessions, and −3.4 at T2 and −6.5 for patients receiving 4 sessions. The same pattern occurred for partners receiving 5 to 8 sessions with a mean reduction of −5.9 at T2 and −4.8 at T3 compared to receiving fewer sessions ranging from a mean reduction from −3.8 to −2.2 at T2 and −4.7 to 1.0 at T3.

Patients and partners had a median of 27 and 24, respectively, on the “Bond” subscale of Working Alliance Inventory—Short Revised. These results substantiate that most patients and partners had a strong therapeutic alliance with the psychologist. However, there was not enough variance among respondents to perform mediation analyses.

A total of 54 patients (56%) in the control group and 53 patients (52%) in the intervention group reported that they had received additional professional support (trial psychologists not included). This was 20 (26%) for partners in the control group and 33 (38%) for partners in the intervention group. This difference was mainly due to perceived support from nurses.

## 4 DISCUSSION

This study did not confirm that a psychological attachment‐oriented couple intervention could further decrease cancer‐related distress than usual care. At T3, we found a significant effect of the intervention on dyadic adjustment for both patients and partners, while partners in the control group had a significant decrease in symptoms of depression.

The nonsignificant findings on cancer‐related distress should be compared to similar studies. A German study including 72 couples coping with BC concluded that the significant differences in cancer‐related distress were caused by baseline differences and not by a differential effect of the couple intervention.^29^ These findings are in line with the results of our study that found a significant effect on distress at T2 for patients (*P* = .05), although not significant when adjusting for baseline values (*P* = .08). The steady decrease in cancer‐related distress in the control group and the significant positive effect for partners on symptoms of depression at T3 in the control group suggest that the patients and partners can cope with both general and cancer‐related distress in the context of usual care.

The significant effect on dyadic adjustment at T3 is in line with other couple intervention studies that found an effect on dyadic adjustment or marital satisfaction.^30^, ^31^ The German RCT of 72 couples found a larger albeit nonsignificant improvement in relationship satisfaction in the intervention group.^29^ The divergent findings might partly be due to different conceptualisations of dyadic adjustment, eg, marital quality or relationship satisfaction.

The results on the primary outcome may be influenced by attrition (attrition rate: 26%‐27% at T3), as indicated by an Australian couple‐based study.^27^ In our study, attrition could affect results into a more positive direction, if participants did not complete the questionnaires due to distress. The opposite might be the case if participants that did not complete the questionnaires did not feel burdened by the BC and found no reason to further participation. However, an analysis found no larger degree of cancer‐related distress in dropouts compared to complete cases at baseline. Though, the significant effect on dyadic adjustment at T3 with no significant effect at T2 may be a chance finding due to attrition.

Our results could not confirm previous studies' recommendations that psychological couple interventions for cancer patients and partners should be offered during the early treatment phase.^32^ We found that 92 of 250 couples (37%), whom had given consent to receive further information regarding the study, declined because they found it difficult to cope with a psychological intervention at that time point (Figure 1). The timing may be more appropriate for partners, because both relationship challenges and challenges related to trying to offer support are present for them while patients are overwhelmed by disease‐related concerns.^33^, ^34^

Study strengths include the randomised controlled design, the large sample size of 198 couples at baseline, the specified primary outcome, and the theoretical framework. Further, the intervention was developed specifically for our sample, the multicentre design increased the generalisability, and the effects of the intervention were investigated in both patients and partners. Timing and content of the couple sessions were adapted for each couple within the limit of 4 to 8 sessions up to 5 months after primary surgery. Further, data on therapeutic alliance showed that the vast majority of couples perceived an alliance with the psychologist, which is an important prerequisite for a successful intervention.^35^, ^36^ We had access to detailed clinical information on each eligible case, which made it possible to adjust for baseline differences in patients' clinical situation.

### 4.1 Study limitations

There is a risk of selection bias in the enrolment procedures followed at the centres. To ensure homogeneity in the screening of and information to eligible patients, clinical staff received written guidelines and coaching with the project manager. Further, attrition throughout the study may have influenced the results, and the initial participation rate of 26% may have decreased the generalisability of the findings. There was no active control group to secure that any effects would be due to the attachment‐oriented intervention and not merely due to attention from a psychologist. Finally, only 53 couples (52%) completed 4 to 8 couple sessions. However, empirical data obtained by trial psychologists suggested that expanding the period for couple sessions for more than 5 months would increase number of couple sessions.

### 4.2 Clinical implications

This study adds important knowledge to the field of couple interventions in cancer. The results suggest that cancer patients and partners generally have a steady decrease in distress over time within the context of usual care. The effect on dyadic adjustment for both patients and partners should be investigated further, to enhance the focus on patients and partners as a dyad in clinical care. It would be interesting to investigate whether increased dyadic adjustment contributes to reduced cancer‐related distress in the re‐entry phase, being the phase, in which patients have to make the transition from treatment to early survivorship.

## CONFLICT OF INTEREST

The authors declare that they have no conflict of interest.

## Supporting information

Appendix S1: Supporting information

## ENDNOTES

*
Breast Surgery Department, Ringsted Hospital

†
Breast Surgery Centre, Plastic Surgery Department, Odense University Hospital

‡
Breast Surgery Department, Herlev Hospital, University of Copenhagen

## References

[1] Rottmann N , Hansen DG , Hagedoorn M , et al. Depressive symptom trajectories in women affected by breast cancer and their male partners: a nationwide prospective cohort study. J Cancer Surviv. 2016;10(5):915‐926. https://doi.org/10.1007/s11764‐016‐0538‐3 27084710 10.1007/s11764-016-0538-3

[2] Bleiker EM , Pouwer F , van der Ploeg HM , Leer JW , Ader HJ . Psychological distress two years after diagnosis of breast cancer: frequency and prediction. Patient Educ Couns. 2000;40(3):209‐217. https://doi.org/10.1016/S0738‐3991(99)00085‐3 10838000 10.1016/s0738-3991(99)00085-3

[3] Christensen S , Zachariae R , Jensen AB , et al. Prevalence and risk of depressive symptoms 3‐4 months post‐surgery in a nationwide cohort study of Danish women treated for early stage breast‐cancer. Breast Cancer Res Treat. 2009;113(2):339‐355. https://doi.org/10.1007/s10549‐008‐9920‐9 18278553 10.1007/s10549-008-9920-9

[4] Henselmans I , Helgeson VS , Seltman H , de Vries J , Sanderman R , Ranchor AV . Identification and prediction of distress trajectories in the first year after a breast cancer diagnosis. Health Psychol. 2010;29(2):160‐168. https://doi.org/10.1037/a0017806 20230089 10.1037/a0017806

[5] Hinnen C , Ranchor AV , Sanderman R , Snijders TAB , Hagedoorn M , Coyne JC . Course of distress in breast cancer patients, their partners, and matched control couples. Ann Behav Med. 2008;36(2):141‐148. https://doi.org/10.1007/s12160‐008‐9061‐8 18797979 10.1007/s12160-008-9061-8

[6] Pistrang N , Barker C . The partner relationship in psychological response to breast cancer. Soc Sci Med. 1995;40(6):789‐797. https://doi.org/10.1016/0277‐9536(94)00136‐H 7747213 10.1016/0277-9536(94)00136-h

[7] Sjovall K , Attner B , Lithman T , et al. Influence on the health of the partner affected by tumor disease in the wife or husband based on a population‐based register study of cancer in Sweden. J Clin Oncol. 2009;27(28):4781‐4786. https://doi.org/10.1200/JCO.2008.21.6788 19720912 10.1200/JCO.2008.21.6788

[8] Fletcher KA , Lewis FM , Haberman MR . Cancer‐related concerns of spouses of women with breast cancer. Psycho‐Oncology. 2010;19(10):1094‐1101. https://doi.org/10.1002/pon.1665 20014184 10.1002/pon.1665 PMC2891846

[9] Fergus KD , Gray RE . Relationship vulnerabilities during breast cancer: patient and partner perspectives. Psycho‐Oncology. 2009;18(12):1311‐1322. https://doi.org/10.1002/pon.1555 19353517 10.1002/pon.1555

[10] Northouse LL , Templin T , Mood D , Oberst M . Couples adjustment to breast cancer and benign breast disease: a longitudinal analysis. Psycho‐Oncology. 1998;7(1):37‐48. https://doi.org/10.1002/(SICI)1099‐1611(199801/02)7:1<37::AID‐PON314>3.0.CO;2‐# 9516649 10.1002/(SICI)1099-1611(199801/02)7:1<37::AID-PON314>3.0.CO;2-#

[11] Pielage SB , Luteijn F , Arrindell WA . Adult attachment, intimacy and psychological distress in a clinical and community sample. Clin Psychol Psychother. 2005;12(6):455‐464. https://doi.org/10.1002/cpp.472

[12] Waldrop DP , O'Connor TL , Trabold N . Waiting for the other shoe to drop: distress and coping during and after treatment for breast cancer. J Psychosoc Oncol. 2011;29(4):450‐473. https://doi.org/10.1080/07347332.2011.582638 21966727

[13] Badr H , Krebs P . A systematic review and meta‐analysis of psychosocial interventions for couples coping with cancer. Psycho‐Oncology. 2013;22(8):1688‐1704. https://doi.org/10.1002/pon.3200 23045191 10.1002/pon.3200 PMC3562417

[14] Brandao T , Schulz MS , Matos PM . Psychological intervention with couples coping with breast cancer: a systematic review. Psychol Health. 2014;29(5):491‐516. https://doi.org/10.1080/08870446.2013.859257 24279379 10.1080/08870446.2013.859257

[15] Regan T , Lambert S , Girgis A , Kelly B , Kayser K , Turner J . Do couple‐based interventions make a difference for couples affected by cancer?: a systematic review. BMC Cancer. 2012;12(1):279 https://doi.org/10.1186/1471‐2407‐12‐279 22769228 10.1186/1471-2407-12-279 PMC3464780

[16] Burwell SR , Brucker PS , Shields CG . Attachment behaviors and proximity‐seeking in cancer patients and their partners. J Couple Relatsh Ther. 2006;5(3):1‐16.

[17] Shaver PR , Mikulincer M , Lavy S , Cassidy J . Understanding and altering hurt feelings: an attachment‐theoretical perspective on the generation and regulation of emotions *In* Feeling Hurt in Close Relationships, Vangelisti AL (ed). 2009 p. 92‐120. doi:https://doi.org/10.1300/J398v05n03_01, 5, 3

[18] Nissen KG . Correlates of self‐rated attachment in patients with cancer and their caregivers: a systematic review and meta‐analysis. Psycho‐Oncology. 2016;25(9):1017‐1027. https://doi.org/10.1002/pon.4057 26763738 10.1002/pon.4057

[19] Hazan C , Shaver PR . Deeper into attachment theory. Psychol Inq. 1994;5(1):68‐79. https://doi.org/10.1207/s15327965pli0501_15

[20] Nicolaisen A , Hansen DG , Hagedoorn M , et al. Attachment‐oriented psychological intervention for couples facing breast cancer: protocol of a randomised controlled trial. BMC Psychology. 2014;2(19). https://doi.org/10.1186/2050‐7283‐2‐19 10.1186/2050-7283-2-19 PMC4363354 25815190

[21] Horowitz M , Wilner N , Alvarez W . Impact of Event Scale: a measure of subjective stress. Psychosom Med. 1979;41(3):209‐218. https://doi.org/10.1192/bjp.180.3.205 472086 10.1097/00006842-197905000-00004

[22] Zigmond AS , Snaith RP . The Hospital Anxiety and Depression Scale. Acta Psychiatr Scand. 1983;67(6):361‐370. https://doi.org/10.1111/j.1600‐0447.1983.tb09716.x 6880820 10.1111/j.1600-0447.1983.tb09716.x

[23] Busby DM , Christensen C , Crane DR , Larson JH . A revision of the dyadic adjustment scale for use with distressed and nondistressed couples: construct hierachy and multidimensional scales. J Marital Fam Ther. 1995;21(3):289‐308. https://doi.org/10.1111/j.1752‐0606.1995.tb00163.x

[24] Munder T , Wilmers F , Leonhart R , Linster H , Barth J . Working Alliance Inventory‐Short Revised (WAI‐SR): psychometric properties in outpatients and inpatients. Clin Psychol Psychother. 2010;17(3):231‐239. https://doi.org/10.1002/cpp.658 20013760 10.1002/cpp.658

[25] Moller S , Jensen MB , Ejlertsen B , et al. The clinical database and the treatment guidelines of the Danish Breast Cancer Cooperative Group (DBCG); its 30‐years experience and future promise. Acta Oncol. 2008;47(4):506‐524. https://doi.org/10.1080/02841860802059259 18465317 10.1080/02841860802059259

[26] Thygesen LC , Daasnes C , Thaulow I , Bronnum‐Hansen H . Introduction to Danish (nationwide) registers on health and social issues: structure, access, legislation, and archiving. Scand J Public Health. 2011;39(7 Suppl):12‐16. https://doi.org/10.1177/1403494811399956 21898916 10.1177/1403494811399956

[27] Scott JL , Halford WK , Ward BG . United we stand? The effects of a couple‐coping intervention on adjustment to early stage breast or gynecological cancer. J Consult Clin Psychol. 2004;72(6):1122‐1135. https://doi.org/10.1037/0022‐006X.72.6.1122 15612858 10.1037/0022-006X.72.6.1122

[28] Manne SL , Ostroff JS , Winkel G , et al. Couple‐focused group intervention for women with early stage breast cancer. J Consult Clin Psychol. 2005;73(4):634‐646. https://doi.org/10.1002/9780470975176.ch13 16173851 10.1037/0022-006X.73.4.634

[29] Heinrichs N , Zimmermann T , Huber B , Herschbach P , Russell D , Baucom D . Cancer distress reduction with a couple‐based skills training: a randomized controlled trial. Ann Behav Med. 2012;43(2):239‐252. https://doi.org/10.1007/s12160‐011‐9314‐9 22037965 10.1007/s12160-011-9314-9

[30] McLean LM , Walton T , Rodin G , Esplen MJ , Jones JM . A couple‐based intervention for patients and caregivers facing end‐stage cancer: outcomes of a randomized controlled trial. Psycho‐Oncology. 2013;22(1):28‐38. https://doi.org/10.1002/pon.2046 21919119 10.1002/pon.2046

[31] Lewis FM , Cochrane BB , Fletcher KA , et al. Helping Her Heal: a pilot study of an educational counseling intervention for spouses of women with breast cancer. Psycho‐Oncology. 2008;17(2):131‐137. https://doi.org/10.1002/pon.1203 17429834 10.1002/pon.1203

[32] Vos PJ , Visser AP , Garssen B , Duivenvoorden HJ , de Haes HCJM . Effects of delayed psychosocial interventions versus early psychosocial interventions for women with early stage breast cancer. Patient Educ Couns. 2006;60(2):212‐219. https://doi.org/10.1016/j.pec.2005.01.006 16442463 10.1016/j.pec.2005.01.006

[33] Harrow A , Wells M , Barbour RS , Cable S . Ambiguity and uncertainty: the ongoing concerns of male partners of women treated for breast cancer. Eur J Oncol Nurs. 2008;12(4):349‐356. https://doi.org/10.1016/j.ejon.2008.04.009 18547865 10.1016/j.ejon.2008.04.009

[34] Hilton BA , Crawford JA , Tarko MA . Men's perspectives on individual and family coping with their wives' breast cancer and chemotherapy. West J Nurs Res. 2000;22(4):438‐459. https://doi.org/10.1177/019394590002200405 10826253 10.1177/019394590002200405

[35] Garfield R . The therapeutic alliance in couples therapy: clinical considerations. Fam Process. 2004;43(4):457‐465. https://doi.org/10.1111/j.1545‐5300.2004.00034.x 15605978 10.1111/j.1545-5300.2004.00034.x

[36] Rait DS . The therapeutic alliance in couples and family therapy. J Clin Psychol. 2000;56(2):211‐224. 10718604 10.1002/(sici)1097-4679(200002)56:2<211::aid-jclp7>3.0.co;2-h

