# Supplementary material for: The effect of an attachment‐oriented couple intervention for breast cancer patients and partners in the early treatment phase: A randomised controlled trial
Source: Psychooncology. 2018 Jan 26;27(3):922–8. doi: 10.1002/pon.4613 (PMC5873374; doi:10.1002/pon.4613)
Supplement: Supplementary file 1 — Appendix S1: Supporting information [file PON-27-922-s001.docx]

Appendix S1

**The Hand in Hand (HiH) intervention**

The theoretical framework of the intervention was attachment theory [1[. The trial psychologists were instructed to explore how each couples’ level of attachment security and proximity, and their attachment behavior (e.g. self-disclosure, expressing empathy, criticism, distancing) [2[ influenced their dyadic coping. Emotion Focused Therapy (EFT) inspired the intervention. The focus of EFT is to reshape negative interactions in couples and to create new emotional experiences that increase a sense of attachment security [3[. The HiH intervention also focused on creating new emotional experiences, but was distinguished from EFT by focusing on BC as the main distressing factor imposing additional individual and dyadic distress. The focus of the HiH intervention was to support couples in engaging in dyadic coping by addressing the impact of attachment style and attachment behavior on how they perceive and offer proximity and security. The intervention was designed to address both initially distressed and non-distressed individuals and couples, because the focus was on breast cancer and the additional strain as a stressor that initiates attachment behavior and increases the risk of experiencing individual and dyadic distress.

**Format of couple sessions**

Psychologists and couples in conformity scheduled sessions. The first session lasted 90 minutes and the following sessions 60 minutes. Sessions were only conducted with attendance of both the patient and partner [1[. The psychologists had no baseline or medical information about the participants. Couple sessions were both conducted at centers and in private practices. If couples randomized to the intervention group did not want to schedule the first session at their first contact with a psychologist, they could call back and schedule it within two months after randomization.

To avoid that participants in the control group received counselling by trial psychologists outside the study, or that participants in the intervention group received more than eight couple sessions, the trial psychologists could not be consulted outside the study until the 10-month follow-up. Regardless of allocation status, all participants were free to consult other psychologists during the time of study.

**Intervention guide**

The project manager and three of the trial psychologists developed the intervention guide. The manual comprised a general introduction to the background, the aim of the intervention and seven issues that should be addressed during the couple sessions [1[. These issues were:

- **Attachment-related security**: Trial psychologists should address couples’ sense of attachment-related security and proximity. This would increase a mutual understanding of attachment behavior and how it affects interdependent coping, and could create new emotional experiences in relation to the challenges that the BC diagnosis imposes on the couple.
- **Individual distress**: Level of individual cancer-related and general distress should be addressed to support the couple in verbalizing their emotions and their perception of security and proximity and identify individual distress that could originate from couple distress.
- **Knowledge and experiences with cancer**: Knowledge and experiences with cancer should be addressed, since previous experiences and expectations can influence attachment behavior and thereby the individual and dyadic adjustment to their current situation.
- **Psychological disorders**: Psychological disorders in any of the participants should be addressed. Depression and anxiety are positively correlated with an insecure attachment style [3[, and present psychological disorders would affect the attachment behavior and the ability to manage distress and engage in interdependent coping.
- **Former stressful life events**: Former stressful life events should be addressed in order to clarify how the couple had adjusted to and managed distress previously. Thereby it was possible to identify what could affect their attachment behavior and interdependent coping in their current situation. The psychologist could support couples that had negative experiences in disclosing their emotions related to these to create a mutual understanding of attachment behavior and support the couple in engaging in interdependent coping related to their current situation. Couples with positive experiences should be supported in continuing their coping strategies.
- **Intimacy and sexual function**: Intimacy and sexual function should be addressed. Individuals with a high level of attachment-related avoidance may be uncomfortable with intimacy [5[. Intimacy is an important aspect of interdependent coping and increases emotion regulation and thereby the management of distress [6[. Discomfort with intimacy would affect the attachment behavior and affect the level of interdependent coping. The role of the psychologist in these situations was to support the couple reach a mutual understanding of their attachment behavior to disclose how their degree of intimacy affected each of them and create a mutual understanding. Sexual functioning is well-known to be affected by a BC diagnosis due to side effects of the treatment. Insecure attachment style has been proved to promote negative feelings related to sexual activity, while secure attachment more often engage in mutual satisfying sexual activities [7[.
- **Other stressors**: Other stressors such as the reactions from own children and other relatives, financial problems and work-related strain should be addressed, since they could add additional distress that would increase the need for proximity and security.

It was stressed that the couple sessions should promote a safe and secure environment [8;9[ in which the couples could create new emotional experiences. In order to do so, the psychologist should address feelings of attachment insecurity, subconscious suppression and rumination of threats [10[.

**Trial psychologists**

The selection of trial psychologists were based on their psychological approach and experience.

They should have an existentialistic approach as receiving a cancer diagnosis can cause great psychological strain and lead to existentialistic reflections. Further, they should integrate attachment-related issues and not defining a right way of dyadic coping, but supporting the couples to dyadic coping that would strengthen their perceived proximity, security and supporting them in creating new emotional experiences. Trial psychologists should be experienced in counselling about normal reactions to a cancer diagnosis for both patients and partners and in identifying relational needs.

Trial psychologists should accept to adhere to the intervention guide but received no additional training with regard to the intervention. They completed a form after each couple session, indicating whether focus had been on the patient, the partner and/or the couple, and which emotions and problems had been addressed in the session. The project manager and the trial psychologist met twice during the study to discuss aspects of the intervention and to reflect upon challenges that occurred. Moreover, the trial psychologists could also have individual discussions with the trial manager regarding this.

**References**

1. Nicolaisen, A., Hansen, D.G., Hagedoorn, M., Flyger, H.E., Rottmann, N., Nielsen, P., Søe, K., Pedersen, A.E., & Johansen, C. 2014. **Attachment-oriented psychological intervention for couples facing breast cancer: protocol of a randomised controlled trial**. BMC Psychology, 2, (19).
2. Stephanie R. Burwell PhD, LMFT , Penny S. Bracker PhD & Cleveland G.

Shields PhD. 2006. **Attachment Behaviors and Proximity-Seeking in Cancer Patients and Their**

**Partners, Journal of Couple & Relationship Therapy**, 5:3, 1-16.

1. Johnson SM, Greenman PS. **The path to a secure bond: Emotionally focused couple therapy**. Journal of Clinical Psychology 2006 May;62(5):597-609.
2. Mikulincer M, Shaver P. **Attachment processes and emotion regulation**. Attachment in adulthood: structure, dynamics and change. 1 ed. New York: The Guilford Press; 2007. p. 188-218.
3. Cobb RJ, Davila J. **Internal working models and change**. In: Obegi JH, Berant E, editors. Attchment theory and research in clinical work with adults. 1 ed. New York: The Guilford Press; 2009. p. 209-33.
4. Berant E, Obegi JH. **Attachment-informed psychotherapy research with adults**. In: Obegi JH, Berant E, editors. Attachment theory and research in clinical work with adults. 1 ed. New York: The Guilford Press; 2009. p. 461-89.
5. Mikulincer M, Shaver P. **Attachment and sex**. Attachment in adulthood: structure, dynamics and change. 1 ed. New York: The Guilford Press; 2007. p. 346-66.
6. Garfield R. **The Therapeutic Alliance in Couples Therapy: Clinical Considerations**. Family Process 2004 Dec;43(4):457-65.
7. Milberg A, Wåhlberg R, Jakobsson M, Olsson EC, Olsson M, Friedrichsen M. **What is a ´secure base` when death is approaching? A study applying attachment theory to adult patients' and family members' experiences of palliative home care**. Psycho-Oncology 2011.
8. Shaver PR, Mikulincer M, Lavy S, Cassidy J. **Understanding and Altering Hurt Feelings: An Attachment-theoretical Perspective on the Generation and Regulation of Emotions**. In: Vangelisti AL, editor. Feeling Hurt in Close Relationships. 2009. p. 92-120.
